# Connecting data, tools and people across Europe: ELIXIR’s response to the COVID-19 pandemic

**DOI:** 10.1038/s41431-020-0637-5

**Published:** 2020-05-15

**Authors:** Niklas Blomberg, Katharina B. Lauer

**Affiliations:** ELIXIR Wellcome Genome Campus Hinxton, Cambridge, CB10 1SD UK

**Keywords:** Genetics research, Social sciences

## Abstract

ELIXIR, the European research infrastructure for life science data, provides open access to data, tools and workflows in the response to the COVID-19 pandemic. ELIXIR’s 23 nodes have reacted swiftly to support researchers in their combined efforts against the pandemic setting out three joint priorities: 1. Connecting national COVID-19 data platforms to create federated European COVID-19 Data Spaces; 2. Fostering good data management to make COVID-19 data open, FAIR and reusable over the long term; 3. Providing open tools, workflows and computational resources to drive reproducible and collaborative science. ELIXIR’s strategy is based on the support given by our national nodes - collectively spanning over 200 institutes - to research projects and on partnering with community initiatives to drive development and adoption of good data practice and community driven standards. ELIXIR Nodes provide support activities locally and internationally, from provisioning compute capabilities to helping collect viral sequence data from hospitals. Some Nodes have prioritised access to their national cloud and compute facilities for all COVID-19 research projects, while others have developed tools to search, access and share all data related to the pandemic in a national healthcare setting.

## Introduction

Open data play a major role in the global response to large-scale outbreaks [1, 2]. For instance, analysis of the 2016 Ebola outbreak points to the importance of open data, including genomic data to generate learnings about diseases where effective vaccines are lacking or have not been developed. The report also points to the role of national response centres as critical arbiters of data access during emergencies [3]. Similarly, open data played a major role in the response to the 2016 Zika outbreak with a commitment from leading national agencies and science organisations to share data [4, 5].

In the current coronavirus outbreak, the global community is mounting a large-scale public health response to understand the pathogen, SARS-CoV-2, and combat coronavirus disease 2019 (COVID-19). Vast amounts of new data are being generated and need to be analysed in the context of pre-existing data and shared for solution-centred research approaches to combat the disease and inform public health policies [6–9].

ELIXIR is the European Life Science Data Infrastructure that brings together life science resources from national centres across Europe. These resources include databases, software tools, training materials, cloud storage and supercomputers. The goal of ELIXIR is to coordinate these resources to form a single infrastructure—accessible across Europe and making it easier for scientists around the globe to find and share data, exchange expertise, and agree on best practices. ELIXIR is a distributed organisation with Nodes in its 22 member states and at EMBL–EBI.

ELIXIR’s priority for the European COVID-19 response is to drive open and rapid access to data, tools and workflows. We will achieve this via the alignment of national infrastructures, European research institutes (e.g. EMBL–EBI) and Horizon 2020 projects including the European Open Science Cloud (EOSC). ELIXIR’s response builds on established standards and working solutions—the already existing collaborations between institutes and national infrastructures. Using these established interfaces enables an agile response and by strenghtening connections between national centres the solution does not only address  immediate needs but also supports the development of a federated European data ecosystem. In this way both learnings and technical solutions will ensure preparedness for the next outbreak.

ELIXIR strives for openness and opportunities for communities to channel efforts into sustainable repositories and registries. ELIXIR’s response rests on three pillars:

1. Federating existing and emerging platforms to connect European COVID-19 data spaces.
2. Fostering good data management practice to make COVID-19 data open, FAIR and reusable over the long-term.
3. Providing open source tools, workflows and computational resources to support reusable and reproducible computational analysis of COVID-19 data.

Below we briefly outline the response—including actions taken by national Nodes—and note that this strategy directly answers to priority action 8 (“Access to research infrastructures”) and action 9 (“Research data sharing platform”) in the EU action plan for COVID-19 [10]. The pandemic is rapidly evolving and so any response needs a great deal of flexibility. ELIXIR provides updated information on services run by ELIXIR Nodes that can be used by researchers working on COVID-19 via elixir-europe.org/services/covid-19-resources.

## Pillar 1: federating existing and emerging platforms to connect European COVID-19 data spaces

ELIXIR’s strategy builds on the developing COVID-19 actions across our member states and supports the convergence of these into Interconnected European Data Spaces for COVID-19 research. By rooting our response in existing national and European infrastructures the strategy contributes to the development of a European data platform for health-related information exchange.

Many, if not most, European countries are planning to collect human sequences and associated phenotype/clinical outcome data in relation to COVID-19 to inform genetic association studies for host factors that determine susceptibility and severity of disease. This will result in rich datasets, e.g. genetics, proteomics and serology, being collected from a large number of COVID-19 patients and these data need to be stored in a secure and GDPR-compliant manner. Thus, there is an urgent demand for data sharing for human host genetic and phenotypic data (from COVID-19 patients within healthcare).

### Create Federated European Genome-Phenome Archives for transnational access of COVID-19 patient data

National infrastructures provide important focal points for the efforts around COVID-19 and many ELIXIR Nodes are tasked with coordinating national data spaces for health, clinical and epidemiological data. The national infrastructures will in many (most) cases provide storage and data management support for national projects. National infrastructures also provide the bulk of data processing capabilities for the many COVID-19 research projects now underway. ELIXIR Nodes are involved in—and in several cases coordinate—these national genomic and phenotypic data stores. The European Genome-phenome Archive (EGA) is a resource for the secure archival and access to all types of potentially identifiable genetic and phenotypic data resulting from biomedical research projects [11]. The EGA is currently transitioning from a centralised model to becoming a federated resource with data deposition and access nodes across Europe. This federated model provides the coordination across Europe to ensure consistent discovery and access to relevant human genetic datasets for research. As much of the COVID-19 host data will come directly from clinical care it will likely be subject to national laws and regulations and therefore, for example, much of the data is unlikely to be consented to leave national jurisdictions. As part of the COVID-19 response ELIXIR will accelerate implementation of the Federated European Genome-phenome Archive (FEGA) to provide the necessary infrastructure and coordination between European national initiatives and ensure rapid sharing of COVID-19 host data in full compliance with national regulations (e.g. GDPR). In addition, the rapid response to the COVID-19 pandemic provides a blueprint for European data access in projects such as 1 Million European Genomes [12].

The federated EGA nodes forms a set of connected European COVID-19 Data Spaces, that bring together national health informatics infrastructure [13] and European archives such as those at EMBL–EBI [14]. This interconnected ecosystem will allow data from ongoing European projects as well as the many re-focussed national research programmes to be widely accessed, reused and linked to global COVID-19 initiatives. Linking of data resources also facilitates research aimed at understanding the origin of pandemics and prevents the risk of future outbreaks: better knowledge on virus–hosts relations also in wild compartments and data supporting “one health approaches”.

## Pillar 2: foster good data management practice to make COVID-19 data open, FAIR and reusable over the long-term

The research response to the COVID-19 outbreak is multifaceted, spanning the development of new therapeutics, drug repurposing, clinical studies to e.g. provide data on epidemiological characteristics, host susceptibility and host immune responses, risk factors for severe disease and routes of transmission. Collectively, the launched national and European projects present a major data generation effort. Ensuring that data are available for access across teams and countries is imperative for the rapid response to the COVID-19 outbreak. ELIXIR will also work with communities to remove obstacles to efficient sharing, for instance the technical interoperability of datasets. A key point for reuse is the quality of the metadata annotation. Many ELIXIR nodes provide data management support to projects launched nationally and at the EU level such that data are published for broad access and reuse.

A particular challenge is data from healthcare providers (“medical data”) and other actors in the healthcare systems (“real-world data”). Discovering, accessing and linking such data is imperative in the response to COVID-19—and extraordinarily challenging, as data from the healthcare systems are encoded with many different standards and governance models. National infrastructures will—as part of the COVID-19 response—engage with the many European stakeholders and ELIXIR will seek to further the development and application of normalisation and interoperability of medical and real-world data by seeking collaborations with projects such as IMI EDHEN and FAIR4Health.

### Support access and reuse of COVID-19 datasets

As a response to the COVID-19 pandemic there is an open, collaborative spirit in the international research community where scientists are offering data and results openly. ELIXIR actively supports these collaborative efforts, e.g. by providing resources for the Global COVID19 BioHackathon (https://github.com/virtual-biohackathons/covid-19-bh20/wiki) to create opportunities to link data and resources and help channel community efforts into long-term sustainable infrastructures.

Public databases are key to ensuring that datasets—from short-read sequence data to protein structures—are stored over the long-term and accessed by scientists in academia and industry. Open science promotes equitable access, globally, and drives reproducibility and long-term sustainability of scientific resources. ELIXIR is an open data infrastructure and advocates the open sharing—wherever possible for reasons of personal privacy and public health—of COVID-19 data. ELIXIR’s response aims to ensure that COVID-19 data are well annotated and accessible for reuse by the research community and society. We encourage researchers to deposit their data in ELIXIR’s Deposition Databases^1^ and in particular all raw sequence data should be deposited in the European Nucleotide Archive (ENA). EMBL–EBI has a dedicated page to assist deposition and sharing of SARS-CoV-2 data into molecular databases.^2^ However, not all data can be shared freely and immediately—in addition to preserving trust and respecting the privacy of patients and participants there might also be public health considerations. Here the federated EGA, where ELIXIR nodes are contributing, will be important, enabling data storage compliant with GDPR. In the first weeks of teh European response ELIXIR has further reached out to the data generation underway in the facilities of the ESFRI Research Infrastructures and ensure that data is deposited in existing archives where possible (e.g. EMBL–EBI COVID19 Portal, ENA, IDR, Biostudies etc.) and are made FAIR for the research community at large.

## Pillar 3: provide open source tools, workflows and computational resources to support reusable and reproducible computational analysis of COVID-19 data

The research community needs, now and for a possible future second wave of the outbreak, reproducible and open-access analysis methods and protocols to progress efficiently. ELIXIR will drive the development and publication of open reproducible tools and workflows for COVID-19 research.

### Roll-out open source tools, workflows and computational resources to support reusable and reproducible computational analysis of COVID-19 data

Galaxy (covid19.galaxyproject.org) is emerging as a key system for connecting national facilities and rapidly deploying open reproducible workflows in the COVID-19 response. In addition, a rapidly increasing number of national facilities are providing cloud compute capacity that underpin the community use of Galaxy. For instance by providing interfaces into open-access workflow and analysis environments (e.g. usegalaxy.eu) and fast-track a minimal viable product for the WorkflowHub.eu, to provide a registry to collect COVID-19 related workflows to ensure that researchers can collaborate, publish and reuse developed workflows and pipelines.

National infrastructures—some of which already form part of EOSC—are also providing open-access biocomputing resources with COVID-19 workflows and toolkits. Infrastructures such as these are open to everyone and are set up to be easily deployed on any cloud infrastructure. Via the EOSC-Life project^3^ we are also investigating if resources can be made available to resource-poor countries in the fight against COVID-19. Specifically, we have reached out to H3Africa Bionet [15] to understand how e.g. usegalaxy.eu can provide additional support for African researchers.

## Working with the community—ELIXIR will catalogue and connect on-going and emerging community, national and global responses to COVID-19 outbreak

European countries are mounting an unprecedented large-scale public health response to cope with the COVID-19 pandemic. Research efforts are rapidly being re-focussed to understand the pathogen, SARS-CoV-2, and combat the disease it causes. The ELIXIR response will support the vast amount of COVID-19 research efforts underway and addresses:

- Connecting national infrastructures and data resources.
- Access to cloud and storage resources for COVID-19 research.
- Collaborative development of open, reusable and reproducible computational workflows.
- Annotation and deposition of data to ascertain reuse and long-term value of these investments

**Fig. 1:**
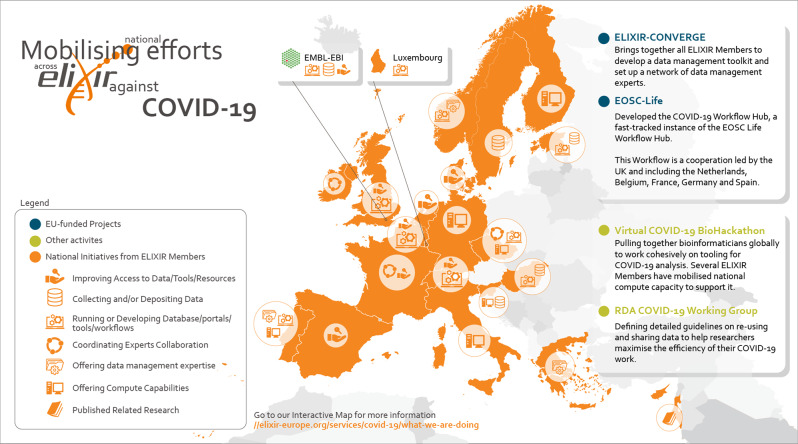
ELIXIR’s drive open and rapid access to data, tools and workflows for the European COVID-19 response. We will achieve this by developing COVID-19 actions across our member states and support the linking of these into Interconnected European Data Spaces for COVID-19. By rooting our response in existing national and European infrastructures the strategy contributes to the development of a European data platform for health-related information exchange. The European landscape is rapidly developing and an up-to-date version of the map is provided online: https://elixir-europe.org/services/covid-19-resources.

As projects on COVID-19 research studies emerge from many initiatives driven from ELIXIR Nodes, ELIXIR has launched the ELIXIR support for SARS-CoV-2 research webpage to display the services that ELIXIR Nodes offer to scientists (Fig. 1). The webpage focuses on Node services that enable European researchers to carry out research into COVID-19. The overview summary provided on this webpage is regularly updated.

Research communities are also coalescing around bottom-up efforts to provide and link COVID-19 data. For example, the virtual COVID-19-BH-20 BioHackathon pulled together bioinformaticians globally to create a cohesive effort and work on tooling for COVID-19 analysis. Many ELIXIR Nodes are supporting this BioHackathon with e.g. cloud compute and storage resources and ELIXIR will continue to work with appropriate community initiatives to help connect researcher-driven initiatives with the emerging European data spaces.

In summary, the ELIXIR strategy responds to the COVID-19 directed actions across our member states and align these actions with the development of Interconnected European Data Spaces for COVID-19. Healthcare is a national—or often regional—competence and a coordinated federated environment for secure data archival, access, and analysis is essential for European collaborations. By rooting our response in existing national and European infrastructures the strategy contributes to the development of a European data platform for health-related information exchange. Via national infrastructures ELIXIR will also drive development and publication of open reproducible tools and workflows for COVID research. The sustainability of the solutions is key—whilst scientific efforts are quickly refocused to support the developing pandemic Europe also needs to think about the longer term. Resources are finite and so investments need to be focused on solutions that can be redeployed towards future challenges and ensure a long-term preparedness.

## Footnotes

1 https://elixir-europe.org/platforms/data/elixir-deposition-databases

2 https://ena-browser-docs.readthedocs.io/en/latest/help_and_guides/sars-cov-2-submissions.html

3 https:/www.eosc-life.eu
